# Clinical features and outcomes of patients with myositis associated-interstitial lung disease

**DOI:** 10.3389/fmed.2022.1096203

**Published:** 2023-01-09

**Authors:** Theodoros Karampitsakos, Vasilios Tzilas, Ourania Papaioannou, Serafeim Chrysikos, Eirini Vasarmidi, Pierre-Antoine Juge, Styliani Vizirianaki, Eleni Bibaki, Argyro Reppa, Prodromos Sidiropoulos, Matthaios Katsaras, Vasilina Sotiropoulou, Panagiota Tsiri, Electra Koulousousa, Eva Theochari, Georgios Tsirikos, Ioannis Christopoulos, Elli Malakounidou, Eirini Zarkadi, Fotios Sampsonas, Georgios Hillas, Theofanis Karageorgas, Dimitrios Daoussis, Christina Kalogeropoulou, Katerina Dimakou, Nikolaos Tzanakis, Raphael Borie, Philippe Dieudé, Katerina Antoniou, Bruno Crestani, Demosthenes Bouros, Argyris Tzouvelekis

**Affiliations:** ^1^Department of Respiratory Medicine, University Hospital of Patras, Patras, Greece; ^2^5th Department of Pneumonology, General Hospital for Thoracic Diseases Sotiria, Athens, Greece; ^3^Laboratory of Molecular and Cellular Pneumonology, Department of Thoracic Medicine, Medical School, University of Crete, Heraklion, Greece; ^4^Université de Paris, INSERM UMR 1152, F-75018, Paris, France; ^5^Assistance Publique – Hôpitaux de Paris (APHP), Service de Pneumologie A, Centre de Référence Constitutif des Maladies Pulmonaires Rares, Fédération Hospitalo-Universitaire (FHU) APOLLO, Hôpital Bichat, Paris, France; ^6^Assistance Publique – Hôpitaux de Paris (APHP), Service de Rheumatologie, Hôpital Bichat-Claude Bernard, F-75018, Paris, France; ^7^Department of Rheumatology, Medical School, University of Crete, Heraklion, Greece; ^8^Department of Rheumatology, Attikon University Hospital, Athens Medical School, National and Kapodistrian University of Athens, Athens, Greece; ^9^Department of Rheumatology, University Hospital of Patras, University of Patras Medical School, Patras, Greece; ^10^Department of Radiology, University Hospital of Patras, Patras, Greece; ^11^First Academic Department of Pneumonology, Hospital for Thoracic Diseases, “SOTIRIA”, Medical School, National and Kapodistrian University of Athens, Athens, Greece

**Keywords:** myositis, interstitial lung disease, organizing pneumonia, amyopathic, survival

## Abstract

**Introduction:**

Myositis associated interstitial lung disease (ILD) seems to be an under-recognized entity.

**Methods:**

In this multicenter, retrospective study, we recorded between 9/12/2019 and 30/9/2021 consecutive patients who presented in five different ILD centers from two European countries (Greece, France) and received a multidisciplinary diagnosis of myositis associated-ILD. The primary outcome was all-cause mortality over 1 year in specific subgroups of patients. Secondary outcomes included comparison of disease characteristics between patients diagnosed with the amyopathic subtype and patients with evidence of myopathy at diagnosis.

**Results:**

We identified 75 patients with myositis associated-ILD. Median age (95% CI) at the time of diagnosis was 64.0 (61.0–65.0) years. Antinuclear antibody testing was positive in 40% of the cohort (*n* = 30/75). Myopathy onset occurred first in 40.0% of cases (*n* = 30), ILD without evidence of myopathy occurred in 29 patients (38.7%), while 16 patients (21.3%) were diagnosed concomitantly with ILD and myopathy. The commonest radiographic pattern was cellular non-specific interstitial pneumonia (NSIP) and was observed in 29 patients (38.7%). The radiographic pattern of organizing pneumonia was significantly more common in patients diagnosed with the amyopathic subtype compared to patients that presented with myopathy [24.1% (*n* = 7/29) vs. 6.5% (*n* = 3/46), *p* = 0.03]. One year survival was 86.7% in the overall population. Kaplan–Meier analysis demonstrated significantly higher all-cause 1-year mortality in patients with the amyopathic subtype compared to patients with evidence of myopathy [H R 4.24 (95% CI: 1.16–15.54), *p* = 0.03]. Patients diagnosed following hospitalization due to acute respiratory failure experienced increased risk of 1-year all-cause mortality compared to patients diagnosed in outpatient setting [HR 6.70 (95% CI: 1.19–37.81), *p* = 0.03]. Finally, patients with positive anti-MDA5 presented with higher 1-year all-cause mortality compared to anti-MDA5 negative patients [HR 28.37 (95% CI: 5.13–157.01), *p* = 0.0001].

**Conclusion:**

Specific ILD radiographic patterns such as NSIP and organizing pneumonia may herald underlying inflammatory myopathies. Hospitalized patients presenting with bilateral organizing pneumonia refractory to antibiotics should be meticulously evaluated for myositis associated-ILD even if there is no overt muscular involvement. Incorporation of ILD radiological patterns in the diagnostic criteria of inflammatory myopathies may lead to timely therapeutic interventions and positively impact patients’ survival.

## Introduction

Idiopathic inflammatory myopathies (IIMs) or “myositis spectrum disorders” comprise a heterogeneous group of systemic autoimmune diseases characterized by varying degree of skeletal muscle inflammation (1). The most commonly used classification involves three main subtypes of IIMs and specifically dermatomyositis, polymyositis and clinically amyopathic dermatomyositis (CADM) (2). The subgroups of IIM have varied in recent years as knowledge on serotype-phenotype correlations has evolved (3). Clinical features of IIMs vary considerably and may include proximal muscle weakness, skin rash, and extramuscular manifestations such as arthralgia, Raynaud’s phenomenon, fever, cardiac arrhythmias, ventricular dysfunction, and interstitial lung disease (ILD). Multiple IIMs phenotypes have been described including overlap syndrome, antisynthetase syndrome, immune mediated necrotizing myopathy and inclusion body myositis. (4–6).

Myositis-associated ILD represents one of the most common extramuscular manifestations occurring in 20–80% of patients with IIMs (7, 8). Importantly, ILD can precede clinically evident muscle or skin disease in a considerable proportion of patients (9–11). Of note, absence of concomitant skin or muscle disease can confound early disease identification and hamper timely interventions leading thus to increased mortality, especially in centers with no expertise in connective tissue disease associated-ILD (9). The need for meticulous evaluation of patients admitted with rapidly progressive respiratory failure seems amenable, given that rapidly progressive-ILD (RP-ILDs) associated with IIMs can be refractory to immunosuppression and result in patients’ admission to the intensive care unit (ICU) (9, 12–14). Negative prognostic indicators include anti-MDA5 antibody positivity, absence of myopathy, older age, and skin ulceration (8, 15–17).

Despite the considerable progress on the knowledge of IIMs phenotypes (3, 18–21) and the advent of recent registries from different parts of the world (1, 22), there is still a pressing need of data for amyopathic cases. This multicenter study aims to present features of patients with myositis associated-ILD from two European countries, compare patients with myopathy and patients with the amyopathic subtype and increase awareness for this under-recognized entity.

## Materials and methods

### Study design and patient selection

In this multicenter, retrospective study, we recorded between 9/12/2019 and 30/9/2021 consecutive patients who presented in five different ILD centers from two European countries (Greece, France) and received a multidisciplinary diagnosis of myositis associated-ILD. Diagnosis was typically set following multidisciplinary discussion, thus muscle biopsy was not always performed. Patients with a follow-up of at least 1 year were included in the analysis. Data collection and analysis was approved by the Institutional Review Board and the Local Ethics Committee (protocol number 28746/9-12-2019).

Age, smoking history, comorbidities, most commonly encountered antibodies, predominant radiographic pattern, functional indices including Forced Vital Capacity% predicted (FVC% pred) and diffusing capacity of the lung for carbon monoxide% predicted (DLCO% pred), cytologic features of bronchoalveolar lavage (BAL), treatment modalities applied, and survival data. Radiographic patterns were reviewed by a radiologist (CK). Antibody profile was obtained using commercially available myositis panel tests such as EUROLINE test kit. Results were available no later than 7 days in most cases.

### Outcome measures

The primary outcome was all-cause mortality over 1 year in specific subgroups of patients: (1) patients diagnosed with the amyopathic subtype compared to patients with evidence of myopathy at diagnosis, (2) patients diagnosed following hospitalization due to acute respiratory failure compared to patients diagnosed in outpatient setting, and (3) patients with positive anti-MDA5 compared to anti-MDA5 negative patients. Secondary outcomes included comparison of disease characteristics such as (1) the frequency of encountered autoantibodies and (2) the most common radiographic patterns, between patients diagnosed with the amyopathic subtype and patients with evidence of myopathy at diagnosis.

### Statistical analysis

With regards to baseline data, summary descriptive statistics were generated with categorical data displayed as absolute numbers and relative frequencies. Continuous data were denoted as mean ± standard deviation (SD) or medians with 95% Confidence Interval (95% CI) following Kolmogorov–Smirnov test for normality. The primary outcome was presented with the Kaplan–Meier method and cumulative incidence curves were compared between the pre-specified groups.

## Results

### Baseline characteristics

We included 75 patients with myositis associated-ILD. Baseline characteristics are summarized in Table 1. Common working diagnoses included antisynthetase syndrome (*n* = 43, 57.3%) and CADM (*n* = 29, 38.7%). Median age (95% CI) at the time of diagnosis was 64.0 (61.0–65.0) years. Most patients were female (57.3%, *n* = 43) and ex-smokers (50.7%, *n* = 38). Mean FVC% predicted ± SD and DLCO% predicted ± SD at the time of diagnosis were 76.5 ± 22.3 and 59.1 ± 27.7, respectively. Median percentage (95% CI) of lymphocytes in BAL was 15.5 (6.0–22.7) in the overall population. Median percentage (95% CI) of lymphocytes in BAL was 13.0 (6.0–20.0) in patients with evidence of myopathy and 21.0 (3.0–43.8) in patients with CADM. Creatine phosphokinase was elevated in 46 patients with evidence of myopathy and 4 patients with CADM (total number of patients: *n* = 50, 66.7% of this cohort).

**TABLE 1:** Baseline characteristics.

Characteristics	(*N*, %)
Total number of patients	75
Age median (%95 CI)	64.0 (61.0–65.0)
Male/Female	32 (42.7%)/43 (57.3%)
Current smokers/Ex-smokers/Never smokers	7 (9.3%)/38 (50.7%)/11 (40.0%)
FVC% predicted ± SD	76.5 ± 22.3
DLCO% predicted ± SD	59.1 ± 27.7
BAL Macrophages% median (%95 CI)	64.0 (59.7–75.7)
BAL Lymphocytes% predicted median (%95 CI)	15.5 (6.0–22.7)
BAL Neutrophils% predicted median (%95 CI)	8.0 (3.0–11.0)
BAL Eosinophils% predicted median (%95 CI)	1.0 (0.0–3.0)
ILD onset first	29 (38.7%)
Concomitant diagnosis of ILD/myopathy	16 (21.3%)
Myopathy onset first	30 (40.0%)
Arterial hypertension	28 (37.3%)
Gastroesophageal reflux disease	14 (18.7%)
Hypothyroidism	11 (14.7%)
Diabetes mellitus	9 (12.0%)
Chronic heart disease	9 (12.0%)
Cancer	8 (10.7%)

BAL, bronchoalveolar lavage; CI, confidence interval; DLCO, diffusing capacity of lung for carbon monoxide; FVC, forced vital capacity; ILD, interstitial lung disease; SD, standard deviation.

Myopathy onset occurred first in 30 patients (40.0%), ILD without evidence of myopathy occurred in 29 patients (38.7%), while 16 patients (21.3%) were diagnosed concomitantly with ILD and myopathy. Patients with evidence of myopathy (*n* = 46, 61.3%) had typically proximal muscle weakness, while distal myopathy was observed following disease progression. The percentage of patients diagnosed with IIM at the time point of hospitalization due to acute respiratory failure was significantly higher in CADM compared to the group with evidence of myopathy [31% (*n* = 9/29) vs. 6.5% (*n* = 3/46), *p* = 0.005].

### Autoantibody profiles

Antinuclear antibody testing (ANA) (40.0%, *n* = 30), anti-Jo-1 (26.7%, *n* = 20), anti-Ro-52 (24.0%, *n* = 18), anti-MDA5 (18.7%, *n* = 14), anti-PL-7 (14.7%, *n* = 11), and anti-PL-12 (14.7%, *n* = 11) were the most frequently encountered antibodies in the overall population (Table 2). Anti-Jo-1 (27.6%, *n* = 8), anti-MDA5 (27.6%, *n* = 8), anti-Ro-52 (24.1%, *n* = 7), anti-PL-7 (20.7%, *n* = 6), and anti-OJ (13.8%, *n* = 4) were the most frequently encountered antibodies in patients diagnosed with CADM. Anti-Ku antibodies were significantly more common in patients with myopathy compared to patients with CADM [13.0%, (*n* = 6/46) vs. 0.0% (*n* = 0/29), *p* = 0.04].

**TABLE 2:** Most frequently encountered autoantibodies.

Auto-antibodies	Overall *N* = 75	Amyopathic at diagnosis *N* = 29	Presence of myopathy at diagnosis *N* = 46
ANA	30 (40.0%)	11 (37.9%)	19 (41.3%)
Anti-Jo-1	20 (26.7%)	8 (27.6%)	12 (26.1%)
Anti-Ro-52	18 (24.0%)	7 (24.1%)	11 (23.9%)
Anti-MDA5	14 (18.7%)	8 (27.6%)	6 (13.0%)
Anti-PL-7	11 (14.7%)	6 (20.7%)	5 (10.9%)
Anti-PL-12	11 (14.7%)	3 (10.3%)	8 (17.4%)
Anti-OJ	9 (12.0%)	4 (13.8%)	5 (10.9%)
Anti-Ku	6 (8.0%)	0 (0.0%)	6 (13.0%)
Anti-Mi-2a	4 (5.3%)	3 (10.3%)	1 (2.2%)
Anti-Mi-2b	4 (5.3%)	3 (10.3%)	1 (2.2%)
Anti-NXP2	3 (4.0%)	2 (6.9%)	1 (2.2%)

ANA, antinuclear antibody; Jo-1, Histidyl-tRNA synthetase; MDA5, melanoma differentiation-associated gene 5; Mi-2a, helicase protein-2a; Mi-2b, helicase protein-2b; NXP2, nuclear matrix protein; OJ, Isoleucyl-tRNA synthetase; PL-7, Threonyl-tRNA synthetase antibodies; PL-12, Alanyl-tRNA synthetase.

### Radiographic patterns

**FIGURE 1:**
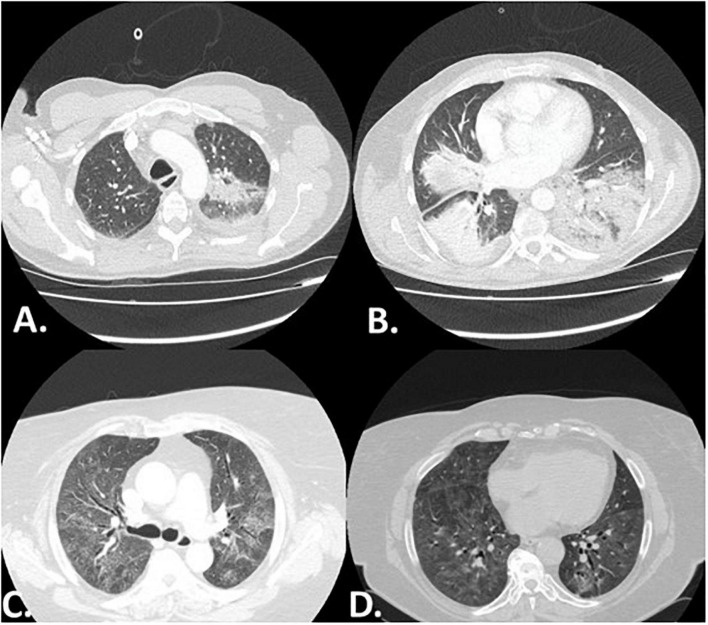
Representative high resolution computed tomography (HRCT) images of two patients with myositis associated-ILD. Panel **(A,B)** depict a patient with RP-ILD presenting with the radiographic pattern of organizing pneumonia. Panel **(C,D)** present a case of myositis in which non-specific interstitial pneumonia (NSIP) was observed 10 years following diagnosis.

The predominant radiographic pattern was non-specific interstitial pneumonia (NSIP) in 46 patients (61.3%), with fibrotic changes in 17 of them (22.7% of the cohort). Organizing pneumonia was observed in 10 patients (13.3%) and NSIP along with organizing pneumonia in 19 patients (25.3%), (Table 3). The radiographic pattern of organizing pneumonia was significantly more common in patients diagnosed with the amyopathic subtype compared to patients that presented with myopathy [24.1% (*n* = 7/29) vs. 6.5% (*n* = 3/46), *p* = 0.03]. Representative images are shown in Figure 1. NSIP was the most common radiographic pattern in patients with anti-Jo-1 [NSIP: 10/20 (50%), NSIP along with organizing pneumonia: 9/20 (45.0%)], anti-PL-7 [NSIP: 8/11 (72.7%), NSIP along with organizing pneumonia: 2/11 (18.2%)], anti-PL-12 [NSIP: 6/11 (54.5%), NSIP along with organizing pneumonia: 3/11 (27.3%)], anti-Ku [NSIP: 6/6 (100%)], and anti-OJ [NSIP: 7/9 (77.8%)] antibodies. Organizing pneumonia was the most common pattern in patients with positive anti-MDA5 [organizing pneumonia: 5/14 (35.7%), organizing pneumonia along with NSIP: 4/14 (28.6%)].

**TABLE 3:** Radiographic patterns identified in the cohort.

Radiographic pattern	Overall *N* = 75	Amyopathic at diagnosis *N* = 29	Presence of myopathy at diagnosis *N* = 46
Cellular NSIP	29 (38.7%)	12 (41.4%)	17 (37.0%)
Overlap NSIP/organizing pneumonia	19 (25.3%)	5 (17.2%)	14 (30.4%)
Fibrotic NSIP	17 (22.7%)	5 (17.2%)	12 (26.1%)
Organizing pneumonia	10 (13.3%)	7 (24.1%)	3 (6.5%)

NSIP, non-specific interstitial pneumonia.

### Treatment modalities

Oral corticosteroids were given to all patients (100%, *n* = 75), with intravenous pulses of methylprednisolone being implemented in 19 patients (25.3%). Other immunosuppressants including rituximab, mycophenolate mofetil, azathioprine, cyclophosphamide, intravenous immune globulin, and methotrexate were applied in 54.7% (*n* = 41), 34.7% (*n* = 26), 20.0% (*n* = 15), 16.0% (*n* = 12), 14.7% (*n* = 11), and 12.0% (*n* = 9) of patients, respectively. Treatment modalities per myositis antibody are summarized in Table 4.

**TABLE 4:** Most common treatment regimens per myositis antibody.

Auto-antibodies	Pulses CS	RTX	MMF	Azathioprine	Cyclophosphamide	IVIG	MTX
Anti-Jo-1	6 (30.0%)	12 (60.0%)	5 (25.0%)	4 (20.0%)	2 (10.0%)	3 (15.0%)	4 (20.0%)
Anti-MDA5	7 (50.0%)	4 (28.6%)	5 (35.7%)	2 (14.3%)	6 (42.9%)	5 (35.7%)	1 (7.1%)
Anti-PL-7	4 (36.4%)	4 (36.4%)	4 (36.4%)	1 (9.1%)	1 (9.1%)	2 (18.2%)	0 (0%)
Anti-PL-12	4 (36.4%)	8 (72.3%)	4 (36.4%)	3 (27.3%)	3 (27.3%)	0 (0%)	0 (0%)
Anti-OJ	2 (22.2%)	6 (54.5%)	2 (22.2%)	2 (22.2%)	1 (11.1%)	0 (0%)	0 (0%)

CS, corticosteroids; Jo-1, Histidyl-tRNA synthetase; IVIG, intravenous immune globulin; MDA5, melanoma differentiation-associated gene 5; MMF, mycophenolate mofetil; MTX, methotrexate; OJ, Isoleucyl-tRNA synthetase; PL-7, Threonyl-tRNA synthetase antibodies; PL-12, Alanyl-tRNA synthetase; RTX, rituximab.

### Survival

**FIGURE 2:**
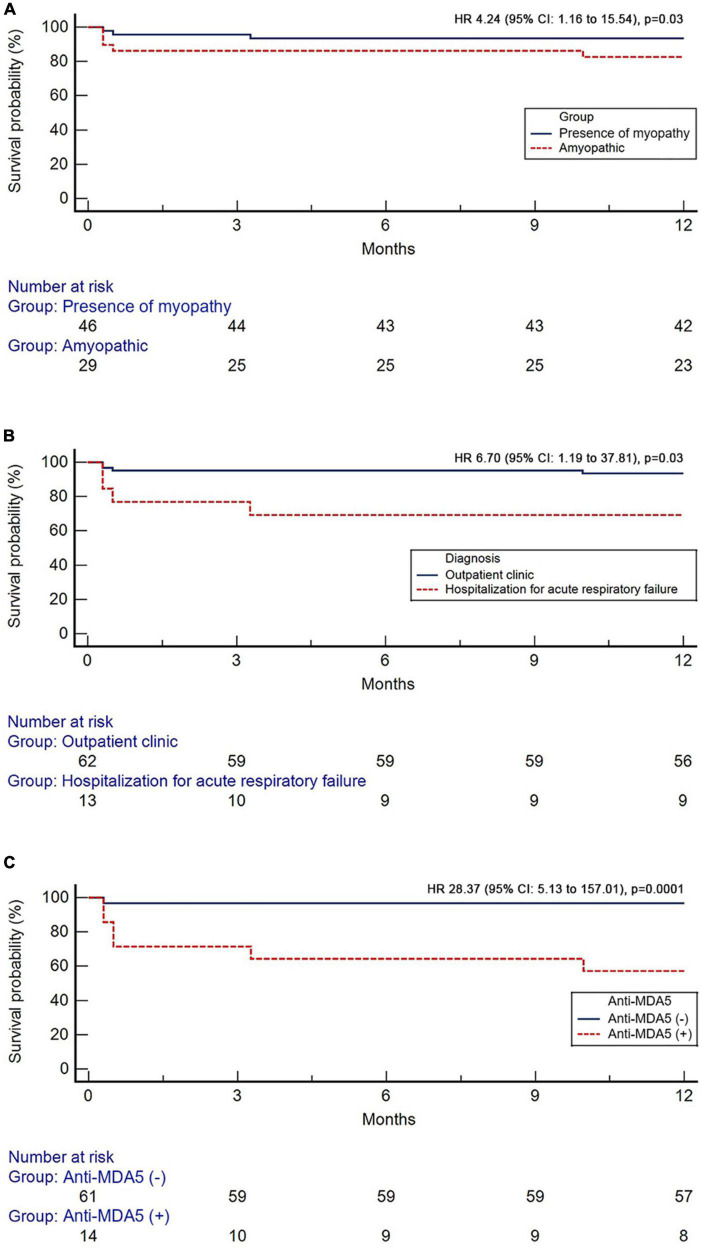
Kaplan–Meier curves showing the prognostic impact of the absence of myopathy **(A)**, diagnosis during hospitalization for acute respiratory failure **(B)**, and anti-MDA5 positivity **(C)**.

One year survival was 86.7% in the overall population. All-cause 1-year mortality was higher in patients with the amyopathic subtype compared to patients with evidence of myopathy [H R 4.24 (95% CI: 1.16–15.54), *p* = 0.03, Kaplan–Meier analysis], (Figure 2A). Patients diagnosed with RP-ILD and acute respiratory failure experienced increased risk of 1-year all-cause mortality compared to patients diagnosed in outpatient setting [HR 6.70 (95% CI: 1.19–37.81), *p* = 0.03], (Figure 2B). Finally, patients with positive anti-MDA5 presented with higher 1-year all-cause mortality compared to anti-MDA5 negative patients [HR 28.37 (95% CI: 5.13–157.01), *p* = 0.0001], (Figure 2C).

## Discussion

This multicenter study demonstrated that ILD involvement in the setting of amyopathic IIM and diagnosis at the time point of hospitalization due to acute respiratory failure are negative prognostic factors in patients with IIM. This study also validated that presence of anti-MDA5 is associated with increased mortality risk. Specific ILD radiographic patterns such as NSIP and organizing pneumonia may herald underlying inflammatory myopathies and most importantly raise the suspicion of autoimmune-associated ILDs with rapidly progressive clinical course requiring timely intervention with immunosuppressants.

The evidence that patients with the amyopathic subtype have worse prognosis represents an important attribute of that study that should be presented upfront. Indeed, a substantial minority of patients presenting in the clinical setting as antibiotic -refractory organizing pneumonia with rapidly-progressing clinical course may display positive myositis-related autoantibodies without any muscle involvement, as assessed by both clinical signs and laboratory parameters (11, 12, 21, 23). This is an important observation as specific ILD radiological patterns including those of organizing pneumonia/NSIP in patients with rapidly progressing respiratory failure may raise the suspicion of an underlying autoimmune-related pneumonitis that will respond to high-doses of corticosteroids and immunosuppressants while escalation of antibiotics will fail, as it happens in our cohort. Unfortunately, in the majority of these cases the conventional serology profile is negative further hampering the diagnosis. To this end, the myositis panel of autoantibodies may unravel the underlying autoimmunity and guide therapeutic decisions.

We showed that diagnosis at the time point of hospitalization due to acute respiratory failure has a negative prognostic impact. Our report couples with previous evidence showing increased mortality risk for myositis-associated RP-ILD (8, 24–26). Myositis-associated RP-ILD is more common in female and younger patients (27). A high index of suspicion is of paramount importance for these rare but treatable cases. Otherwise, these patients are often admitted to ICU due to Acute Respiratory Distress Syndrome (ARDS). Intriguingly, critically ill patients are frequently not evaluated for myositis-associated pulmonary processes, leading thus to excess mortality due to the absence of appropriate treatment. In the multinational Large Observational Study to Understand the Global Impact of Severe Acute Respiratory Failure -LUNG SAFE- cohort, only 12 out of 234 patients with ARDS of unknown etiology had been screened for autoimmune etiology of RP-ILD (28).

Screening for autoimmune etiology in these patients should not be limited to ANA testing. Antisynthetase antibodies are cytoplasmic and subsequently ANA testing is frequently negative in patients with myositis associated-ILD. Therefore, negative ANA should not preclude further testing with myositis-specific antibodies (9, 29). This has been corroborated by our cohort, in which only 40% of patients were ANA positive. Importantly, classification criteria of the European League Against Rheumatism and American College of Rheumatology had been widely criticized for the exclusion of non-Jo-1 antisynthetase antibodies (9, 30). The aforementioned is further corroborated by our cohort in which anti-Jo-1 were positive only in 26.7% of cases. Patients with myositis presented with substantial variability with regards to myositis-specific positive antibodies.

Myositis-specific positive antibodies might have prognostic significance and unravel specific phenotypes based on this study and previous reports (31). Anti-MDA5 positivity is closely associated with CADM and has a negative prognostic role for patients with myositis (8, 12, 15, 32–36). Features including presence of ILD, mucocutaneous or necrotic ulcerations, Gottron papules, painful, and erythematous papules especially over the palmar surfaces should raise suspicion of anti-MDA5 positivity and fuel meticulous immunologic investigation even in the absence of myopathy (37–39). Early identification of anti-MDA5 can lead to timely therapeutic interventions and may positively impact patients’ survival. Several compounds have been used for the management of anti-MDA5 myositis including corticosteroids and steroid sparing agents (40). Most recently, emerging data support the use of tofacitinib in these patients (41, 42). With regards to other autoantibodies, anti-PL7 and anti-PL12 have been characterized as indicators of more severe lung involvement (31). Risk of cancer is elevated in patients with anti- Transcriptional intermediary factor-1γ, Nuclear matrix protein 2 and 3-hydroxy-3-methylglutaryl-CoA reductase antibodies (43). Finally, in our cohort anti-Ku were significantly more common in patients with myopathy.

With regards to radiographic patterns, in consistency with previous studies, most patients presented with NSIP and/or organizing pneumonia (8, 44). Patchy bilateral areas of ground-glass attenuation predominantly localized in the lower lobes along with areas of consolidation, septal thickening and traction bronchiectasis seem to be common radiographic findings in these patients (45). Prominent consolidation refractory to antibiotics is typical of acute onset ILD and responds well to corticosteroids or immunosuppressive compounds if intervention is timely. Presence of ground-glass attenuation combined with reticulation and bronchiectasis is more common in chronic cases potentially underdiagnosed due to lack of extensive immunologic evaluation. Of note, American thoracic society (ATS)/European respiratory society (ERS) statement highlighted that overlap of NSIP and organizing pneumonia or presence of the fibrosing variant of organizing pneumonia are suspicious for underlying myositis (46).

Our study presents with limitations that should be treated cautiously. First, our sample size is relatively moderate and we could not compare the effect of different treatment regimens in particular subgroups; yet, this sample size seems acceptable for this rare entity. Second, we did not present long term follow-up data; nonetheless, our aim was to increase awareness for timely diagnosis and intervention in this under-recognized entity. Finally, this study has the inherent weakness of a retrospective study.

Collectively, this study showed that ILD is the hallmark of pulmonary involvement in both myopathic and amyopathic forms of inflammatory myopathies and may precede myopathy onset. Respiratory physicians should be alert to organizing pneumonia cases without overt muscular involvement. Hospitalized patients presenting with bilateral organizing pneumonia refractory to antibiotics should be meticulously evaluated for myositis associated-ILD as certain autoimmune profiles such as anti-MDA5 have a negative prognostic role for these patients. The term of amyopathic forms of IIMs in the context of RP-ILD is quite often misjudged and questioned by rheumatologists as autoantibodies presenting in the myositis panel may also independently affect the lung interstitium without muscle involvement. To this end, we believe that the term “autoimmune-induced lung injury or ILD” could be most appropriate in this context. Incorporation of myositis-specific ILD radiological patterns and all myositis-specific antibodies in the diagnostic criteria of inflammatory myopathies may lead to timely and effective therapeutic interventions.

## Data availability statement

The raw data supporting the conclusions of this article will be made available by the authors, without undue reservation.

## Ethics statement

Data collection and analysis was approved by the Institutional Review Board and the Local Ethics Committee (protocol number 28746/9-12-2019). The patients/participants provided their written informed consent to participate in this study. Written informed consent was obtained from the individual(s) for the publication of any potentially identifiable images or data included in this article.

## Author contributions

TdK and AT were involved in study conception, data collection, statistical analysis, and drafting of the initial version of the manuscript. VT and OP were involved in data collection and drafting of the manuscript. SC, EV, P-AJ, SV, EB, AR, PS, MK, VS, PT, EK, ET, GT, IC, EM, EZ, FS, GH, TfK, DD, CK, KD, NT, and RB were involved in data collection and offered important intellectual contribution. RB, PD, KA, BC, and DB supervised the work along with AT. DB and AT supervised the work. All authors offered significant intellectual contribution for the last version of the manuscript and approved the final form.

## References

[1] Lilleker J Vencovsky J Wang G Wedderburn L Diederichsen L Schmidt J The EuroMyositis registry: an international collaborative tool to facilitate myositis research. *Ann Rheum Dis.* (2018) 77:30–9. 10.1136/annrheumdis-2017-211868 28855174 PMC5754739

[2] Lundberg I Tjärnlund A Bottai M Werth V Pilkington C Visser M 2017 European League Against Rheumatism/American College of Rheumatology classification criteria for adult and juvenile idiopathic inflammatory myopathies and their major subgroups. *Ann Rheum Dis.* (2017) 76:1955–64. 10.1136/annrheumdis-2017-211468 29079590 PMC5736307

[3] Lundberg I Fujimoto M Vencovsky J Aggarwal R Holmqvist M Christopher-Stine L Idiopathic inflammatory myopathies. *Nat Rev Dis Primers.* (2021) 7:86. 10.1038/s41572-021-00321-x 34857798

[4] Dalakas M. Inflammatory muscle diseases. *New Engl J Med.* (2015) 372:1734–47. 10.1056/NEJMra1402225 25923553

[5] Rider L Miller F. Deciphering the clinical presentations, pathogenesis, and treatment of the idiopathic inflammatory myopathies. *JAMA.* (2011) 305:183–90. 10.1001/jama.2010.1977 21224460 PMC4047218

[6] Cottin V Thivolet-Béjui F Reynaud-Gaubert M Cadranel J Delaval P Ternamian PJ Interstitial lung disease in amyopathic dermatomyositis, dermatomyositis and polymyositis. *Eur Respir J.* (2003) 22:245–50. 10.1183/09031936.03.00026703 12952255

[7] Kiely P Chua F. Interstitial lung disease in inflammatory myopathies: clinical phenotypes and prognosis. *Curr Rheumatol Rep.* (2013) 15:359. 10.1007/s11926-013-0359-6 23888366

[8] Li Y Gao X Li Y Jia X Zhang X Xu Y Predictors and mortality of rapidly progressive interstitial lung disease in patients with idiopathic inflammatory myopathy: A series of 474 patients. *Front Med (Lausanne).* (2020) 7:363. 10.3389/fmed.2020.00363 32850886 PMC7412929

[9] Jablonski R Bhorade S Strek M Dematte J. Recognition and management of myositis-associated rapidly progressive interstitial lung disease. *Chest.* (2020) 158:252–63. 10.1016/j.chest.2020.01.033 32059958

[10] Tzilas V Sfikakis P Bouros D. Antisynthetase syndrome masquerading as hypersensitivity pneumonitis. *Respiration.* (2021) 100:1105–13. 10.1159/000516508 34148050

[11] Trallero-Araguás E Grau-Junyent J Labirua-Iturburu A García-Hernández F Monteagudo-Jiménez M Fraile-Rodriguez G Clinical manifestations and long-term outcome of anti-Jo1 antisynthetase patients in a large cohort of Spanish patients from the GEAS-IIM group. *Semin Arthritis Rheum.* (2016) 46:225–31. 10.1016/j.semarthrit.2016.03.011 27139168

[12] Vuillard C Pineton de Chambrun M de Prost N Guérin C Schmidt M Dargent A Clinical features and outcome of patients with acute respiratory failure revealing anti-synthetase or anti-MDA-5 dermato-pulmonary syndrome: a French multicenter retrospective study. *Ann Intensive Care.* (2018) 8:87. 10.1186/s13613-018-0433-3 30203297 PMC6131681

[13] Morisset J Johnson C Rich E Collard H Lee J. Management of myositis-related interstitial lung disease. *Chest.* (2016) 150:1118–28. 10.1016/j.chest.2016.04.007 27102182

[14] Hervier B Uzunhan Y. Inflammatory myopathy-related interstitial lung disease: From pathophysiology to treatment. *Front Med.* (2019) 6:326. 10.3389/fmed.2019.00326 32010700 PMC6978912

[15] Moghadam-Kia S Oddis C Sato S Kuwana M Aggarwal R. Anti-Melanoma differentiation-associated gene 5 Is associated with rapidly progressive lung disease and poor survival in US patients with amyopathic and myopathic dermatomyositis. *Arthritis Care Res.* (2016) 68:689–94. 10.1002/acr.22728 26414240 PMC4864500

[16] Sato S Masui K Nishina N Kawaguchi Y Kawakami A Tamura M Initial predictors of poor survival in myositis-associated interstitial lung disease: a multicentre cohort of 497 patients. *Rheumatology (Oxford, England).* (2018) 57:1212–21. 10.1093/rheumatology/key060 29596687

[17] Fujiki Y Kotani T Isoda K Ishida T Shoda T Yoshida S Evaluation of clinical prognostic factors for interstitial pneumonia in anti-MDA5 antibody-positive dermatomyositis patients. *Mod Rheumatol.* (2018) 28:133–40. 10.1080/14397595.2017.1318468 28490218

[18] Chen F Wang J Zhang P Zuo Y Ye L Wang G Interstitial lung disease in dermatomyositis without myositis-specific and myositis-associated autoantibodies: Study of a series of 72 patients from a single cohort. *Front Immunol.* (2022) 13:879266. 10.3389/fimmu.2022.879266 35603153 PMC9120579

[19] González-Pérez M Mejía-Hurtado J Pérez-Román D Buendía-Roldán I Mejía M Falfán-Valencia R Evolution of pulmonary function in a cohort of patients with interstitial lung disease and positive for antisynthetase antibodies. *J Rheumatol.* (2020) 47:415–23. 10.3899/jrheum.181141 31203227

[20] Maloir Q Laurence S Christian V Fanny G Renaud L Julien G. Clinical experience in anti-synthetase syndrome: a monocentric retrospective analytical study. *Acta Clin Belg.* (2022) 77:624–30. 10.1080/17843286.2021.1925818 34000974

[21] Wilfong E Young-Glazer J Sohn B Schroeder G Annapureddy N Gillaspie E Anti-tRNA synthetase syndrome interstitial lung disease: A single center experience. *Respir Med.* (2022) 191:106432. 10.1016/j.rmed.2021.106432 33994288 PMC8566329

[22] Feldon M Farhadi P Brunner H Itert L Goldberg B Faiq A Predictors of reduced health-related quality of life in adult patients with idiopathic inflammatory myopathies. *Arthritis Care Res.* (2017) 69:1743–50. 10.1002/acr.23198 28118525 PMC5524619

[23] Suda T Fujisawa T Enomoto N Nakamura Y Inui N Naito T Interstitial lung diseases associated with amyopathic dermatomyositis. *Eur Respir J.* (2006) 28:1005–12. 10.1183/09031936.06.00038806 16837503

[24] Won Huh J Soon Kim D Keun Lee C Yoo B Bum Seo J Kitaichi M Two distinct clinical types of interstitial lung disease associated with polymyositis-dermatomyositis. *Respir Med.* (2007) 101:1761–9. 10.1016/j.rmed.2007.02.017 17428649

[25] Fujisawa T Hozumi H Kono M Enomoto N Hashimoto D Nakamura Y Prognostic factors for myositis-associated interstitial lung disease. *PLoS One.* (2014) 9:e98824. 10.1371/journal.pone.0098824 24905449 PMC4048238

[26] Tillie-Leblond I Wislez M Valeyre D Crestani B Rabbat A Israel-Biet D Interstitial lung disease and anti-Jo-1 antibodies: difference between acute and gradual onset. *Thorax.* (2008) 63:53–9. 10.1136/thx.2006.069237 17557770

[27] Shi J Li S Yang H Zhang Y Peng Q Lu X Clinical profiles and prognosis of patients with distinct antisynthetase autoantibodies. *J Rheumatol.* (2017) 44:1051–7. 10.3899/jrheum.161480 28461650

[28] de Prost N Pham T Carteaux G Mekontso Dessap A Brun-Buisson C Fan E Etiologies, diagnostic work-up and outcomes of acute respiratory distress syndrome with no common risk factor: a prospective multicenter study. *Ann Intensive Care.* (2017) 7:69. 10.1186/s13613-017-0281-6 28631088 PMC5476531

[29] Ashton C Paramalingam S Stevenson B Brusch A Needham M. Idiopathic inflammatory myopathies: a review. *Internal Med J.* (2021) 51:845–52. 10.1111/imj.15358 34155760

[30] Lundberg I Tjärnlund A Bottai M Werth V Pilkington C Visser M 2017 european league against rheumatism/american college of rheumatology classification criteria for adult and juvenile idiopathic inflammatory myopathies and their major subgroups. *Arthritis Rheumatol.* (2017) 69:2271–82. 10.1002/art.40320 29106061 PMC5846474

[31] Pinal-Fernandez I Casal-Dominguez M Huapaya J Albayda J Paik J Johnson C A longitudinal cohort study of the anti-synthetase syndrome: increased severity of interstitial lung disease in black patients and patients with anti-PL7 and anti-PL12 autoantibodies. *Rheumatology (Oxford).* (2017) 56:999–1007. 10.1093/rheumatology/kex021 28339994 PMC5850781

[32] Hozumi H Enomoto N Kono M Fujisawa T Inui N Nakamura Y Prognostic significance of anti-aminoacyl-tRNA synthetase antibodies in polymyositis/dermatomyositis-associated interstitial lung disease: a retrospective case control study. *PLoS One.* (2015) 10:e0120313. 10.1371/journal.pone.0120313 25789468 PMC4366175

[33] Kishaba T McGill R Nei Y Ibuki S Momose M Nishiyama K Clinical characteristics of dermatomyosits/polymyositis associated interstitial lung disease according to the autoantibody. *J Med Invest.* (2018) 65:251–7. 10.2152/jmi.65.251 30282869

[34] Yoshida N Okamoto M Kaieda S Fujimoto K Ebata T Tajiri M Association of anti-aminoacyl-transfer RNA synthetase antibody and anti-melanoma differentiation-associated gene 5 antibody with the therapeutic response of polymyositis/dermatomyositis-associated interstitial lung disease. *Respir Invest.* (2017) 55:24–32. 10.1016/j.resinv.2016.08.007 28012490

[35] Tanizawa K Handa T Nakashima R Kubo T Hosono Y Aihara K The prognostic value of HRCT in myositis-associated interstitial lung disease. *Respir Med.* (2013) 107:745–52. 10.1016/j.rmed.2013.01.014 23485097

[36] Allenbach Y Uzunhan Y Toquet S Leroux G Gallay L Marquet A Different phenotypes in dermatomyositis associated with anti-MDA5 antibody: Study of 121 cases. *Neurology.* (2020) 95:e70–8. 10.1212/wnl.0000000000009727 32487712 PMC7371381

[37] Charrow A Vleugels R. Cutaneous Ulcerations in Anti-MDA5 Dermatomyositis. *New Engl J Med.* (2019) 381:465. 10.1056/NEJMicm1816147 31365803

[38] Fiorentino D Chung L Zwerner J Rosen A Casciola-Rosen L. The mucocutaneous and systemic phenotype of dermatomyositis patients with antibodies to MDA5 (CADM-140): a retrospective study. *J Am Acad Dermatol.* (2011) 65:25–34. 10.1016/j.jaad.2010.09.016 21531040 PMC3167687

[39] Tzouvelekis A Karampitsakos T Bouros E Tzilas V Liossis S Bouros D. Autoimmune biomarkers, antibodies, and immunologic evaluation of the patient with fibrotic lung disease. *Clin Chest Med.* (2019) 40:679–91. 10.1016/j.ccm.2019.06.002 31376900

[40] Karampitsakos T Vraka A Bouros D Liossis S Tzouvelekis A. Biologic treatments in interstitial lung diseases. *Front Med.* (2019) 6:41. 10.3389/fmed.2019.00041 30931306 PMC6425869

[41] Chen Z Wang X Ye S. Tofacitinib in amyopathic dermatomyositis-associated interstitial lung disease. *New Engl J Med.* (2019) 381:291–3. 10.1056/NEJMc1900045 31314977

[42] Kurasawa K Arai S Namiki Y Tanaka A Takamura Y Owada T Tofacitinib for refractory interstitial lung diseases in anti-melanoma differentiation-associated 5 gene antibody-positive dermatomyositis. *Rheumatology (Oxford, England).* (2018) 57:2114–9. 10.1093/rheumatology/key188 30060040

[43] Benveniste O Stenzel W Allenbach Y. Advances in serological diagnostics of inflammatory myopathies. *Curr Opin Neurol.* (2016) 29:662–73. 10.1097/wco.0000000000000376 27538058

[44] Yura H Sakamoto N Satoh M Ishimoto H Hanaka T Ito C Clinical characteristics of patients with anti-aminoacyl-tRNA synthetase antibody positive idiopathic interstitial pneumonia. *Respir Med.* (2017) 132:189–94. 10.1016/j.rmed.2017.10.020 29229096

[45] Lega J Reynaud Q Belot A Fabien N Durieu I Cottin V. Idiopathic inflammatory myopathies and the lung. *Eur Respir Rev.* (2015) 24:216–38. 10.1183/16000617.00002015 26028634 PMC9487811

[46] Travis W Costabel U Hansell D King T Jr. Lynch D Nicholson A An official american thoracic society/european respiratory society statement: Update of the international multidisciplinary classification of the idiopathic interstitial pneumonias. *Am J Respir Crit Care Med.* (2013) 188:733–48. 10.1164/rccm.201308-1483ST 24032382 PMC5803655

